# A study of endothelial function and circulating asymmetric dimethylarginine levels in people with Type 1 diabetes without macrovascular disease or microalbuminuria

**DOI:** 10.1186/1475-2840-8-27

**Published:** 2009-06-01

**Authors:** Latika Sibal, Sharad C Agarwal, Edzard Schwedhelm, Nicole Lüneburg, Rainer H Böger, Philip D Home

**Affiliations:** 1 Newcastle University, Newcastle upon Tyne, UK; 2 Institute of Experimental and Clinical Pharmacology and Toxicology, University Medical Center Hamburg-Eppendorf, Hamburg, Germany

## Abstract

**Background:**

Asymmetric dimethylarginine (ADMA) is a competitive inhibitor of endothelial nitric oxide synthase (eNOS) that is associated with endothelial dysfunction, and is a risk marker for cardiovascular disease, a significant problem in Type 1 diabetes. The aim of the present study was to measure circulating ADMA, and define its association with endothelial dysfunction and endothelial markers in people with Type 1 diabetes with low likelihood of macrovascular disease.

**Methods:**

Sixty-one young people with Type 1 diabetes without macrovascular disease or nephropathy and 62 healthy volunteers underwent brachial artery flow-mediated dilatation (FMD) and assay of plasma ADMA and adhesion molecules.

**Results:**

Age, gender, BMI, lipid profile and renal function were similar in the two groups. People with Type 1 diabetes had impaired FMD compared to healthy controls (5.0 ± 0.4 vs 8.9 ± 0.4%; p < 0.001). Plasma ADMA levels were significantly lower in the people with diabetes compared to healthy controls (0.52 ± 0.12 vs 0.66 ± 0.20 μmol/l, p < 0.001). Plasma ICAM-1, E-selectin and PAI-1 levels were significantly higher in people with diabetes compared to healthy controls (median 201 (IQR 172–226) vs 180 (156–216) μg/l, p = 0.027; 44.2 (32.6–60.9) vs. 33.1 (22.4–51.0) μg/l; p = 0.003 and 70.8 (33.3–85.5) vs 46.3 (23.9–76.8) μg/l, p = 0.035). Plasma ADMA and VCAM-1 levels were positively correlated (r = 0.37, p = 0.003) in people with diabetes. There was no correlation between the plasma ADMA and FMD.

**Conclusion:**

ADMA levels are not associated with endothelial dysfunction in young adults with Type 1 diabetes without microalbuminuria or known macrovascular disease. This suggests that the impaired endothelial function in these individuals is not a result of eNOS inhibition by ADMA.

## Background

Type 1 diabetes is associated with endothelial dysfunction and increased cardiovascular risk [1]. Endothelial nitric oxide synthase (eNOS) converts L-Arginine to nitric oxide (NO), which is a key mediator of vascular homeostasis due to its central role in the maintenance of the endothelial milieu. ADMA is a competitive inhibitor of eNOS, which thus reduces the production of NO and might possibly cause endothelial dysfunction [2]. The circulating levels of ADMA have been found to be raised in the presence of cardiovascular risk factors including hypertension, renal dysfunction and Type 2 diabetes as well as in individuals with cardiovascular disease [3-6]. Studies which assessed ADMA levels in people with Type 1 diabetes have reported conflicting results [7,8].

Circulating ADMA concentration is eliminated in part by enzymatic degradation by dimethylarginine dimethylaminohydrolases (DDAH)-1 and -2, and in part by renal excretion [9,10]. While normally DDAH activity accounts for about 80% of total body elimination of ADMA with renal excretion contributing only 20%, under pathophysiological conditions renal function may have a stronger influence on ADMA levels [11,12]. In Type 1 diabetes this might be true in the earlier stages when renal hyperfiltration prevails, as well as when diabetic nephropathy develops, suggesting that different stages of disease may variably affect ADMA concentrations.

Endothelial function can be modulated by several factors associated with diabetes including degree of acute hyperglycaemia, duration of diabetes, accumulation of advanced glycosylated end products and complications such as nephropathy and microalbuminuria [13]. Endothelial function can be assessed non-invasively by measuring brachial artery flow-mediated dilatation (FMD). Soluble adhesion molecules like intercellular adhesion molecule-1 (ICAM-1), vascular cellular adhesion molecule-1 (VCAM-1) and E-selectins are involved in the recruitment of leucocytes to sites of inflammation at the endothelium and are thus involved in the pathogenesis of atherosclerosis [14]. Plasma plasminogen activator inhibitor-1 (PAI-1) is mainly produced by the endothelium and is the major physiological inhibitor of tissue type plasminogen activation. Elevated PAI-1 levels increase the risk of atherothrombosis and may promote the progression of vascular disease [15].

The underlying mechanism of endothelial dysfunction in Type 1 diabetes is not fully understood. Experimental animal studies have shown that prolonged exposure to hyperglycaemia can cause enhanced eNOS expression with increased NO release but at the same time with an even more profound increase in superoxide anion (O_2_^-^) levels [16].

The aim of the present study was to measure circulating ADMA levels and their association with cellular adhesion molecules, PAI-1 levels, and FMD in people with Type 1 diabetes with low likelihood of arterial wall damage.

## Methods

### Participants

The study population was 61 people with Type 1 diabetes without macrovascular disease or microalbuminuria and 62 healthy volunteers, all age 16–35 years. Type 1 diabetes required serum C-peptide <0.15 nmol/l when plasma glucose >5.5 mmol/l or a history of ketoacidosis with Type 1 diabetes phenotype. All were insulin-treated and had a duration of diabetes of >1 yr. Absence of microalbuminuria was determined by measurement of urinary albumin:creatinine ratio (last three samples all <2.5 mg/mmol in men, <3.5 mg/mmol in women), and of macrovascular disease by absence of history of a cardiovascular event or procedure, angina (Rose questionnaire), ischaemic ECG abnormalities, use of statins or ACE inhibitors, and abnormal pedal pulses. The participants were attending the Newcastle Diabetes Centre for routine diabetes care. The age-matched non-diabetic control group was recruited from relatives, friends and contacts of the participants and investigators, and local offices. They were also screened to exclude the presence or possibility of arterial disease. Local ethical committee approval was obtained from Gateshead and South Tyneside Local Research Ethics Committee. All participants gave written informed consent to participation in the study.

### Procedures

Study procedures were performed at Newcastle Diabetes Centre, and at the Department of Medical Physics, Newcastle General Hospital. Medical examination included a 12-lead ECG, the Rose angina questionnaire, blood pressure measurement whilst sitting by automatic sphygmomanometer (Omron, HEM-773AC, Bannokburn, Illinois, USA), and body weight in indoor clothing without shoes.

Women were studied in the first 10 days of the menstrual cycle. All the participants attended early morning, fasting, having avoided caffeinated beverages, cigarettes and strenuous exercise since the previous evening. They were given a low-fat breakfast, informally matched to usual carbohydrate intake, together with usual insulin dose in those with diabetes. The post-prandial state was chosen to avoid the insulin-deficient state often found in people with Type 1 diabetes at the end of the night, and because of evidence that the post-prandial state is more pathogenetically significant for the development of vascular damage in people with diabetes [17].

### Measurements and assays

#### Flow-mediated dilatation (FMD)

Brachial artery FMD was measured by a trained scientist (L Sibal) using high resolution ultrasound machine (5–12 MHz linear transducer, HDI 5000, ATL, Bothell, WA, USA) with data acquisition by artificial neural network software (VIA software, London, UK) imaging the vessel wall 20 times/sec and in accordance with international guidelines [18,19]. The brachial artery was studied 20–100 mm proximal to the antecubital fossa in supine participants after 15 min rest. Pressure in an upper-forearm sphygmomanometer cuff was raised to 250 mmHg for 5 min, and FMD automatically calculated as the percentage increase in diastolic diameter after reactive hyperaemia 55–65 seconds after deflation to baseline. After a further 15 min, 400 μg sublingual glyceryl trinitrate (GTN) was given, and diastolic diameter remeasured after 5 min for measurement of endothelial-independent dilatation.

#### Biochemical analysis

HbA_1c _was measured using a DCCT-aligned HPLC (Tosoh Bioscience, Stuttgart, Germany) method (normal reference <6.1%). Serum lipids were measured by standard enzymatic methods, with HDL cholesterol by a routine, antibody based homogeneous method. Other biochemical analyses were measured by standard methods in the same clinical trials accredited laboratory (Royal Victoria Infirmary, Newcastle upon Tyne, UK). Estimated glomerular filtration rate (eGFR), using the four-variable MDRD equation was calculated [20]. Plasma plasminogen activator inhibitor-1 (PAI-1) was measured by ELISA, plasma soluble E-selectin (E-selectin), plasma soluble intercellular adhesion molecule-1 (ICAM-1) and plasma soluble vascular cellular adhesion molecule-1 (VCAM-1) by immunoassay by a solid phase ELISA.

Plasma ADMA concentration was determined by an ELISA kit (DLD Diagnostika, Hamburg, Germany) which has recently been described and validated in detail [21]. In brief, cross-reactivity was 1.2% for symmetric dimethylarginine (SDMA) and <0.02% for L-arginine. The limit of detection was 0.05 μmol/l. There is a good correlation of the values measured by this ELISA and LC-tandem MS (n = 29; r = 0.984; p < 0.0001). Reference ranges for ADMA have been established by this technique recently [22].

### Statistical analysis

Statistical analysis was performed using SPSS for Windows (Statistical Package for Social Science, version 15; Chicago, Illinois, USA). Comparisons of the groups were examined by Student's *t *test for normally distributed data, Mann Whitney *U *test for nonparametric data and Chi square test for categorical variables. Pearson and Spearman correlation coefficients were used to determine the relationships between continuous variables. Stepwise regression analysis was performed to assess predictors of ADMA levels adjusted for gender and smoking and including all variables with a p < 0.10. A p value of <0.05 was considered statistically significant for all analyses.

## Results

The characteristics of the participants are shown in Table 1. The mean duration of diabetes was 11.4 ± 6.6 years. The fasting plasma glucose level was 10.0 ± 4.6 mmol/l and the HbA_1c _8.5 ± 1.7% in the people with diabetes, statistically higher than the healthy controls as expected. The lipid profile and the renal functions were not statistically significantly different in the two groups (Table 1).

**Table 1:** Characteristics of the people with Type 1 diabetes and the healthy control group studied

	Type 1 diabetes	Healthy controls	p
n	61	62	
Age (yr)	25.2 ± 5.2	24.6 ± 4.7	0.459
Duration of diabetes (yr)	11.4 ± 6.6	-	
Sex (M/F)	39/35	36/44	0.339
Smoking (n (%))	18 (24)	11 (14)	0.094
BMI (kg/m^2^)	25.1 ± 3.4	24.9 ± 4.2	0.739
Waist-hip ratio	0.89 ± 0.07	0.87 ± 0.06	0.256
Systolic BP (mmHg)	120 ± 12	120 ± 10	0.957
Diastolic BP (mmHg)	71 ± 8	72 ± 10	0.625
HbA_1c _(%)	8.6 ± 1.6	5.2 ± 0.3	<0.001
Fasting plasma glucose (mmol/l)	10.2 ± 4.8	4.8 ± 0.5	<0.001
Post-prandial glucose (mmol/l)	11.2 ± 5.6	4.5 ± 0.8	<0.001
Serum cholesterol (mmol/l)	4.8 ± 0.9	4.5 ± 0.9	0.182
Serum HDL cholesterol (mmol/l)	1.5 ± 0.3	1.5 ± 0.4	0.967
Serum triglyceride (mmol/l)	0.9 (0.7–1.4)	0.9 (0.7–1.3)	0.451
LDL cholesterol (mmol/l)	2.7 ± 0.8	2.6 ± 0.7	0.330
Serum creatinine (μmol/l)	86 ± 13	83 ± 13	0.254
Estimated glomerular filtration rate (eGFR) (ml/min/1.73-m^2^)	90.3 ± 13.7	86.3 ± 15.6	0.263
**Medications (n (%))**			
Metformin	1 (1)	0 (0)	
Thyroxine	5 (7)	0 (0)	
Oral contraceptives	5 (7)	1 (1)	
Antidepressants	2 (3)	0 (0)	
Multivitamins	3 (4)	9 (11)	
Inhalers	0 (0)	6 (8)	

Mean ± SD; Median (IQR); or n(%)

To convert values for glucose to milligrams per decilitre, multiply by 18.01. To convert values for cholesterol to milligrams per decilitre, multiply by 38.67. To convert values for triglycerides to milligrams per decilitre, multiply by 88.57. To convert values for creatinine to milligrams per decilitre, multiply by 0.011.

### Soluble endothelial markers and ADMA levels

Plasma ADMA levels were significantly lower in people with diabetes compared to healthy controls (0.52 ± 0.02 vs 0.66 ± 0.03 μmol/l, p < 0.001) (Table 2). The ADMA levels were comparable amongst the smokers and non-smokers in people with diabetes (0.52 ± 0.03 vs 0.51 ± 0.02 μmol/l, p = 0.868). Furthermore, ADMA levels were comparable in men and women with diabetes (0.52 ± 0.02 vs 0.52 ± 0.02 μmol/l, p = 0.967).

**Table 2:** Measures of endothelial function, ultrasonographic measures, and circulating markers of endothelial dysfunction in people with Type 1 diabetes and healthy controls

	Type 1 diabetes	Healthy controls	p
ADMA (μmol/l)	0.52 ± 0.02	0.66 ± 0.03	<0.001
ICAM-1 (μg/l)	201.3 (171.7–226.4)	179.5 (156.4–216.3)	0.027
VCAM-1 (μg/l)	549.9 (477.2–666.2)	550.5 (471.6–631.3)	0.395
E-Selectin (μg/l)	44.2 (32.6–60.9)	33.1 (22.4–51.0)	0.003
PAI-1 (μg/l)	70.8 (33.3–85.5)	46.3 (23.9–76.8)	0.035
FMD (%)	5.0 ± 0.4	8.9 ± 0.4	<0.001
GTN (%)	16.1 ± 0.8	24.1 ± 1.0	<0.001

Mean ± SE; Median (IQR)

The plasma ICAM-1 levels were significantly higher in people with diabetes compared to healthy controls (median 201 (IQR 172–226) vs 180 (156–216) μg/l, p = 0.027). Plasma PAI-1 levels were significantly higher in the people with diabetes compared to healthy controls (70.8 (33.3–85.5) vs 46.3 (23.9–76.8) μg/l, p = 0.035). Plasma VCAM-1 levels were comparable between the two groups (550 (477–666) vs 551 (472–631) μg/l, p = 0.395) (Table 2).

**Figure 1:**
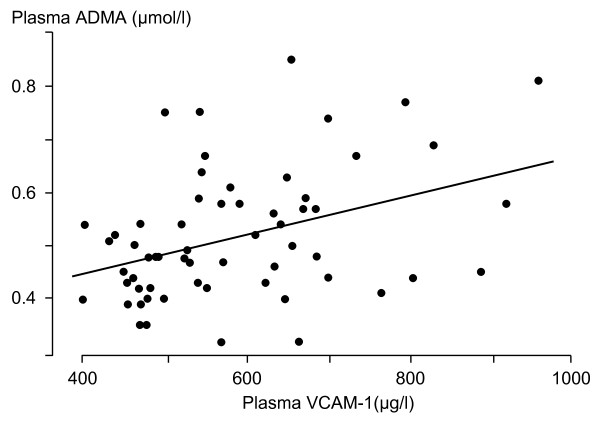
**Relationship between plasma ADMA and VCAM-1 levels in people with Type 1 diabetes (r = 0.37, p = 0.003)**.

In people with diabetes, the plasma PAI-1 levels were positively correlated with serum total cholesterol levels (r = 0.41, p = 0.001), LDL cholesterol (r = 0.266, p = 0.045), triglyceride (r = 0.35, p = 0.006), and with HbA_1c_(r = 0.38, p = 0.003). In people with diabetes, plasma ADMA and VCAM-1 levels were positively correlated (r = 0.37, p = 0.003) (Figure 1). Stepwise regression analysis showed that ADMA was independently predicted by VCAM-1 (standardized beta coefficient 0.40, p = 0.002). Other relationships were not statistically significant.

### Endothelial function

In people with diabetes FMD was significantly impaired compared to healthy controls (5.0 ± 0.4 vs 8.9 ± 0.4%, p < 0.001). Brachial artery vasodilatation in response to GTN was significantly impaired in people with diabetes compared to the healthy controls (16.1 ± 0.8 vs 24.1 ± 1.0%, p < 0.001). There was no correlation between the plasma ADMA levels and the endothelial function.

## Discussion

Type 1 diabetes is associated with increased cardiovascular disease and mortality. Endothelial dysfunction is an early feature in the development of vascular complications in people with diabetes [1]. The present study has found impaired endothelial function, decreased ADMA levels and increased soluble endothelial markers and PAI-1 levels in people with Type 1 diabetes without any evidence of macrovascular complications or microalbuminuria. Our study is consistent with previous studies [8,23] which showed reduced levels of ADMA in individuals with Type 1 and Type 2 diabetes, whilst contrasting with the results of another study reporting higher levels in people with Type 1 diabetes compared to healthy individuals [7].

Hyperglycaemia is associated with endothelial dysfunction both in-vivo [24] and in-vitro [16]. The underlying mechanism of endothelial dysfunction in Type 1 diabetes is not fully understood. Reduced NO levels seem to play a central role in the development of endothelial dysfunction amongst the multiple pathogenetic mechanisms that have been postulated. NO levels might be reduced as a result of a combination of decreased NO production because of decreased activity and/or reduced expression of eNOS, or decreased activity of NO, or its increased degradation by reactive oxygen species or increased production of superoxide ions. A weakness of our study is then that we did not make any measure of oxidative stress.

ADMA has emerged as an independent predictor of cardiovascular risk and is increased in people with risk factors for cardiovascular disease [6]. Studies of ADMA in people with diabetes report conflicting results [7,23]. Our study has shown significantly lower levels of ADMA in people with Type 1 diabetes compared to healthy individuals and a lack of association between endothelial function and ADMA. This probably suggests that the endothelial dysfunction in these individuals is not necessarily due to decreased NO production because of eNOS inhibition by ADMA.

The finding of lower ADMA levels in Type 1 diabetes in our study is consistent with a previous study in children with uncomplicated Type 1 diabetes [8]. Lower levels of ADMA were reported in people with Type 2 diabetes and the ADMA levels were associated with glomerular filtration rate (GFR) but not with risk factors of vasculopathy [23]. We also did not find any association between endothelial function or risk factors for CVD and plasma ADMA levels in the individuals with diabetes. Paiva and colleagues suggested that the lower level of ADMA in diabetic people with no complications could be because of possibly increased excretion of ADMA due to hyperfiltration [23]. Renal vasodilatation and hyperfiltration has been shown in experimental diabetes and in people with early diabetic nephropathy and microalbuminuria [25]. Furthermore, ADMA has been shown to accumulate in people with chronic renal failure [4].

Although there was a trend for eGFR to be higher in people with diabetes than in the healthy people, this difference was not statistically significant. Circulating ADMA concentration is regulated by an interplay between its generation during the hydrolytic breakdown of methylated proteins, enzymatic degradation to L-citrulline and dimethylamine by dimethylarginine dimethylaminohydrolases (DDAH)-1 and -2, and renal excretion [9,10,26]. While normally, DDAH activity accounts for about 80% of total body elimination of ADMA, it is possible that disturbance of GFR by hyperglycaemia could be a factor in lowering ADMA levels. Hyperglycaemia may also directly influence ADMA levels through unknown mechanisms by influencing either the production of ADMA or its metabolism by DDAH.

The results of this present study are in contrast to the study published by Altinova and colleagues who showed plasma ADMA levels to be significantly higher in people with uncomplicated Type 1 diabetes [7]. Notably, the ADMA levels (by HPLC technique) in that study (7) were almost five-to nine-fold higher in the people with diabetes compared to the levels reported in the present study (by ELISA) and by other research groups(HPLC) [23]. However, eGFR was not reported in that study (7). It can be speculated that the higher ADMA in that study could possibly be due to a relatively reduced eGFR in the people with diabetes, though this could be attributed to the different assay technique used. Elevated plasma ADMA levels have been shown to be increased in patients with early diabetic nephropathy which in turn confer increased cardiovascular morbidity [27].

Experimental studies have demonstrated that hyperglycaemia leads to upregulation of eNOS causing increased production of NO by 30–40% in cultured endothelial cells, whilst increasing the superoxide anion production drastically by almost 300% [16]. Interestingly, in vivo studies in streptozotocin- induced diabetic rats have shown up-regulation of eNOS, marked reduction of vascular NO bioavailability as well as endothelial dysfunction [28]. The up-regulated eNOS system can get uncoupled leading to superoxide production rather than NO production. Endothelial NOS (eNOS) enzymatic activity is regulated, amongst other factors, by the availability of co-factor tetrahydrobiopterin (BH4). Diabetes mellitus is associated with decreased BH4 levels and this can lead to uncoupling of eNOS and result in production of superoxide rather than NO [29]. Experimental studies have shown that overexpression of eNOS accelerates atherosclerotic lesion formation in apolipoprotein E-deficient mice lacking sufficient BH4 [30]. In a study in people with hypertension, Dixon and colleagues suggested that eNOS is present in the uncoupled state in people who have coexisting hypertension and diabetes, thus contributing to significantly increased superoxide levels [31]. As ADMA is a competitive inhibitor of eNOS, it may be possible that the decreased ADMA levels in people with uncomplicated Type 1 diabetes might be detrimental to the vascular milieu in these people as this may further facilitate eNOS overexpression and increased superoxide production and cardiovascular risk.

Intercellular adhesion molecule-1 (ICAM-1), E-selectin and PAI-1 levels were significantly increased in the diabetic people and are consistent with previous studies [32,33]. We noted a positive correlation between the PAI-1 levels and the traditional risk factors of increased cardiovascular risk. In a recent study PAI-1 levels independently related to coronary artery calcium, a surrogate for subclinical CVD, in young people with Type 1 diabetes [34]. Plasma ICAM-1 levels have been shown to be associated with several established cardiovascular risk factors [14,35]. We found a positive correlation between plasma VCAM-1 levels and the plasma ADMA levels. Increased ADMA levels have been shown to increase mononuclear cell adhesiveness in hypercholesterolaemic patients [36], an effect that may be mediated by enhanced endothelial expression of VCAM-1 and other adhesion proteins [37].

## Conclusion

In summary, our study has shown that people with Type 1 diabetes uncomplicated with microalbuminuria or macrovascular disease have impaired endothelial function although with lower levels of ADMA. This is associated with increased soluble endothelial markers and PAI-1 levels thus providing a milieu for increased thrombovascular disease. Further studies are required to elucidate the role of ADMA in endothelial dysfunction in people with Type 1 diabetes.

## Abbreviations

(ADMA): Asymmetric dimethylarginine; (FMD): flow-mediated dilatation; (eNOS): endothelial nitric oxide synthase; (NO): nitric oxide; (DDAH): dimethylarginine dimethylaminohydrolases; (ICAM-1): intercellular adhesion molecule-1; (VCAM-1): vascular cellular adhesion molecule-1; (PAI-1): Plasma plasminogen activator inhibitor-1; (O_2_^-^): superoxide anion; (GTN): glyceryl trinitrate; (eGFR): Estimated glomerular filtration rate; (MDRD): Modification of Diet in Renal Disease; (BH4): tetrahydrobiopterin; (CVD): Cardiovascular disease.

## Competing interests

Grants/fellowships supporting the writing of this paper-None.

Disclosure summary-RHB and ES are named as inventors on European Patent 1666884 and receive royalties from these. LS, SCA, NL and PDH have no potential dualities of interest relevant to this article.

## Authors' contributions

LS and PDH participated in the conception and design of the study; LS and SCA participated in the acquisition, analysis, interpretation of data as well as drafting the manuscript; ES, NL participated in revising the manuscript critically; RHB helped to interpret the data, and participated in revising the manuscript critically for intellectual content; PDH also helped to interpret the data, draft the manuscript and revise it.
